# Inflammatory dilated cardiomyopathy: Etiology and clinical management

**DOI:** 10.1007/s00059-020-04900-8

**Published:** 2020-03-02

**Authors:** Bernhard Maisch, Sabine Pankuweit

**Affiliations:** 1 Medical Faculty, Philipps University, Marburg, Germany; 2 Heart and Vessel Centre, Marburg, Germany; 3 Department of Internal Medicine and Cardiology, Philipps University and UKGM, Marburg, Germany

**Keywords:** Myocarditis, Endomyocardial biopsy, Immunohistology, Intravenous immunoglobulins, Immunosuppressive therapy, Myokarditis, Endomyokardbiopsie, Immunhistologie, Intravenöse Immunglobuline, Immunsuppressive Therapie

## Abstract

Inflammatory dilated cardiomyopathy (DCMi) is a syndrome, not an etiological disease entity. The infective etiology and the immunopathology can be best determined through endomyocardial biopsy with a complete work-up by light microscopy, immunohistology, and polymerase chain reaction for microbial agents. This review focuses on the methodological advances in diagnosis in the past few years and exemplifies the importance of an etiology-orientated treatment in different case scenarios. In fulminant nonviral myocarditis, immunosuppressive treatment together with hemodynamic stabilization of the patient via mechanical circulatory support (e.g., microaxial pumps, extracorporeal membrane oxygenation, left ventricular assist device) can be life-saving. For viral inflammatory cardiomyopathy, intravenous immunoglobulin treatment can resolve inflammation and often eradicate the virus.

## Abstract (de)

Die dilatative inflammatorische Kardiomyopathie (DCMi) ist ein Syndrom, keine ätiologische Krankheitsentität. Ihre infektiöse Ursache und Immunpathogenese lässt sich am besten durch die Endomyokardbiopsie und die Aufarbeitung der Biopsieproben mittels klassischer Färbungen für die Lichtmikroskopie, durch Immunhistologie, Immunhistochemie der infiltrierenden Zellen und molekularbiologisch durch die quantitative und qualitative Polymerasekettenreaktion (PCR) für mikrobielle Erreger erfassen. Die vorliegende Übersicht beschränkt sich einerseits auf die methodischen Entwicklungen der letzten Jahre und zeigt andererseits an klinischen Fallbeispielen die Bedeutung eines ätiologischen Therapiekonzepts bei der Versorgung der Patienten. Bei fulminanter, nichtviraler Myokarditis kann sich neben der intensivmedizinischen Versorgung, der hämodynamischen Überbrückung durch mechanische Kreislaufunterstützung, z. B. mit Mikroaxialpumpen, ECMO („extracorporeal membrane oxygenation“, extrakorporale Membranoxygenierung) oder LVAD („left ventricular assist device“, linksventrikuläres Unterstützungssystem), die immunsuppressive Behandlung als lebensrettend erweisen. Bei virusinduzierter inflammatorischer Kardiomyopathie kann eine i.v.-Immunglobulintherapie die Entzündung eliminieren und zur Eradikation des verursachenden Virus beitragen.

Dilated cardiomyopathy (DCM) is a heterogeneous group of myocardial diseases clinically defined by the presence of left ventricular dilatation and contractile dysfunction [1, 2]. Using the broadest definition of DCM would make the disease equivalent to heart failure since it would also include cardiac dysfunction after myocardial infarction and through remodeling. In the Anglo-Saxon literature, ischemic cardiomyopathy is often mentioned. The classic definition of DCM Europe excludes coronary artery disease because its etiology is clear, whereas in idiopathic DCM the cause has yet to be further clarified. As such, a progression from viral myocarditis to DCM has long been hypothesized. Supporting this possibility, studies by endomyocardial biopsy for the definite diagnosis of myocarditis have provided evidence of inflammation and/or viral infection within the myocardium in patients with DCM, thus triggering the initiation or progression of myocarditis to postinflammatory DCM. Complementary to the recent reviews by Imanaka-Yoshida [1] and Maisch [2], this contribution examines molecular and clinical data of the progression from viral and autoreactive (nonviral) myocarditis to DCM and offers perspectives beyond mere heart failure management to a treatment of the underlying cause.

## Diagnostic developments and their implications for clinical diagnosis

### Molecular diagnosis in patients

Myocarditis—a frequent cause of DCM and sudden cardiac death—typically results from cardiotropic viral infection followed by active inflammatory destruction of the myocardium. Advances in the molecular detection of viruses by endomyocardial biopsy have improved our ability to diagnose and understand the pathophysiological mechanisms of this elusive disease; these methods were summarized in 2013 by Klingel and Pankuweit [3]. The following is a condensed summary of their review and will be available in parts in an upcoming Springer textbook titled *Viral Myocarditis: From Experimental Models to Diagnosis in Patients* (Eds. Alida L. P. Caforio).

The diagnosis of virus-associated myocarditis was clearly facilitated by the introduction of endomyocardial biopsy techniques by Sakakibara and Konno in 1962 [4] and the development of polymerase chain reaction (PCR) by Saiki et al. in 1985 [5]. The combination of both methods made it possible, for the first time, to detect viral genomes directly within the affected myocardial tissue in patients with suspected myocarditis. A wide range of different PCR assays have been developed, which are suitable for identifying different cardiac RNA and/or DNA viruses with a higher sensitivity than standard immunohistochemical methods used for the detection of viral proteins [3, 6–9].

Through these molecular approaches, enteroviruses have been identified as highly relevant pathogenic agents in myocarditis [10–18]. Moreover, the presence of genomes from adenoviruses, parvovirus B19 (B19V; [19]), herpesviruses (human herpes virus 6 [HHV6]), cytomegalovirus (CMV), Epstein–Barr virus (EBV), herpes simplex virus type 1 (HSV1; [20]), *Chlamydia pneumoniae* [21], *Borrelia burgdorferi* [22, 23], as well as other infectious agents [24] was reported in patients with inflammatory heart disease.

A problem associated with the analysis of cardiotropic agents by PCR is the fact that this technique allows only for the detection of viral genomes without differentiating potentially infected cardiac cell types. In addition, active replication of the virus is generally not investigated by PCR [25]. Thus, in order to substantiate the etiopathogenetic role of an infectious agent by PCR, the patient’s symptoms and hemodynamics must be carefully evaluated in the context of the histological, immunohistochemical, and molecular findings from the endomyocardial biopsies.

To overcome this diagnostic gap, the in situ hybridization technique was established, which can attribute viral sequences to specific cells types in the heart. Also, as shown for coxsackie viruses, in situ hybridization allows for the detection of viral plus-strand RNA as well as the replicative minus-strand RNA intermediates, which are of particular interest for the diagnosis of active myocardial infections [12–14, 25, 26].

Starting from 2002, fluorescence-based real-time PCR assays were established for the evaluation of the viral load in the heart. Regarding the quantification of parvovirus B19 genomes, real-time PCR assays have been developed for use with the light cycler system [27], fluorescence resonance energy transfer probes [28], as well as for the ABI (Applied Biosystem Inc.) Prism system [29, 30].

### Prevalence of cardiotropic viruses in endomyocardial biopsies assessed by molecular tools

Viral genomes were identified in patients with acute or chronic myocarditis and with DCM, but the impact of these viral genomes on cardiac function and clinical outcome is still debated [31]. The overall prevalence of cardiotropic viruses amplified by reverse transcription (RT)-PCR in endomyocardial biopsies of these patients differs widely: Enteroviral genomes were detected in 3–53%, cytomegalovirus DNA in 3–40%, and adenoviruses in 3–23% of the myocardium of patients with inflammatory heart disease. A wide range of results have been obtained by different molecular methods and an epidemiologic shift in Europe from the infection by entero- and adenoviruses to parvovirus B19 viruses [2].

### Prevalence of parvovirus B19 genomes in patients with myocarditis and DCM

Investigations in adult patients with inflammatory heart diseases revealed a prevalence of parvovirus B19 DNA in 19.5% of patients with myocarditis, 23% in patients with inflammatory dilated cardiomyopathy (DCMi), and 16% in our patients with DCM [32]. Comparable prevalence rates have been confirmed by others [33], who also reported that persistence of parvovirus B19 in patients with left ventricular dysfunction was found to be associated with a progressive impairment of left ventricular ejection fraction, whereas spontaneous viral elimination was associated with a significant improvement in left ventricular function [33]. However, in contrast to enteroviruses, spontaneous virus elimination of parvovirus B19 was observed in only a proportion of patients. These results suggest that persisting cardiac viral infections may constitute a major cause of progressive left ventricular dysfunction in patients with past myocarditis or DCM. In 24 patients who presented with acute-onset angina pectoris and ST-segment elevations or T‑wave inversion mimicking acute myocardial infarction but with normal coronary angiogram, the histological analysis excluded mostly active or borderline myocarditis. But instead B19V, enteroviruses, and adenoviruses genomes were detected in the myocardium of 12, three, and two patients, respectively [34]. In the autopsy study of a female patient with clinical signs of myocardial infarction and histopathological fulminant myocarditis, the presence of B19V genomes was exclusively located in endothelial cells of the smaller intramyocardial vessels. Immunohistochemical staining exhibited marked expression of E‑selectin by endothelial cells, a finding indicative of endothelial dysfunction [35]. These findings may explain the fact that many patients with B19V-associated myocarditis present with the clinical signs of microangiopathy that are also typical of ischemic heart disease.

However, the causal relationship of B19V infections with cardiac disease has been questioned, mainly because epidemiological data demonstrated a lifelong persistence of B19V genomes in various organs, apart from the heart [36, 37], and the fact that B19V DNA was also detected in heart tissue from patients without clinical manifestations of inflammatory cardiomyopathy [38–40].

Nevertheless, parvovirus replication in myocardial endothelial cells was substantiated by the detection of B19V RNA replicative intermediates in the myocardium only of inflamed hearts, whereas viral RNA was not detected in chronic DCM without inflammation or in control hearts [41]. On the basis of these data, it was suggested that viral loads of more than 500 genome equivalents per microgram isolated nucleic acid in endomyocardial biopsies are the clinically relevant threshold for the maintenance of myocardial inflammation.

In a recent publication with human blood samples, endothelial-derived microparticles were significantly different in B19V+ compared with B19V-patients and human controls, with an increase of apoptotic but not activated endothelial microparticles [42], indicating that differences in the subtypes of microparticles can be attributed to specific myocardial viral infections.

However, the molecular mechanisms responsible for a possible reactivation of B19V, the influence of the immune system triggering B19V replication, and immune-independent viral pathogenesis in uninflamed hearts are the remaining gaps in our understanding of B19V pathogenicity in heart diseases [41].

### Prevalence of EBV and human herpesvirus 6 in patients with myocarditis and DCM

In immunocompetent patients, herpesviruses including EBV and HHV6 infections rarely induce cardiac symptoms. EBV-linked acute pericarditis or myocarditis is reported only in a few immunocompetent patients [43, 44], as was HHV6-induced myocarditis in a low number of patients, but sometimes with a fatal outcome [45, 46]. Investigation of autopsy material showed diffuse myocarditis with a granulocytic and monocytic infiltrate, necrotizing arteritis of the coronary arteries, and fulminant hepatitis with microvesicular steatosis and necrosis together with the detection of the HHV6 genome in heart, liver, lung, and spleen [45]. In the larger series of patients with inflammatory heart diseases, analyses for HHV6 and EBV were always included. Prevalence rates for HHV6 genomes detected in patients with myocarditis or DCM ranged from 8 to 20% and for EBV genomes from 0 to 8%. Nevertheless, the pathophysiological mechanisms of herpesviruses in acute myocarditis and especially the possible relevance of HHV‑6 reactivation for the development of chronic cardiomyopathies remain to be assessed.

### Prevalence of influenza virus RNA in patients with myocarditis and DCM

Several cases of acute myocarditis especially in juvenile patients have been reported in association with pandemic H1N1 influenza virus infections. Genomes of influenza A/H1N1 virus were detected by RT-PCR analysis in blood as well as in myocardial tissue in patients [47] and particularly in children [48, 49] with a lethal influenza virus infection.

In larger series of patients with myocarditis and DCM investigated by Kühl et al. [50] and Kandolf et al. [51], it was shown that the detection of two or more cardiotropic viruses by PCR in the myocardium is not uncommon. In 3219 patients with cardiac dysfunction and suspected myocarditis, HHV6 and B19V genomes were concurrently detected in the heart in 11.6% of the 20% of patients with multiple infections [51].

## Diagnostic implications

There is convincing evidence from animal models and investigations in humans that viral infections can induce a significant damage of cardiomyocytes through direct virus-mediated injury and secondary immune reactions, finally leading to chronic myocarditis and DCM [52].

Consequently, the position statement of the European Society of Cardiology Working Group on Myocardial and Pericardial Diseases points out that endomyocardial biopsy is the standard for diagnosing myocarditis and should be performed early in the course of the disease using multiple specimens to optimize diagnostic accuracy and reduce sampling error, especially in focal myocarditis [53–57]. Immunohistochemistry should be performed to demonstrate infiltrating cells by antibodies specific for activated T and B cells, macrophages, major histocompatibility class 1 and class 2 antigens, adhesion molecules, and endothelial cells. Specific binding of the antibodies indicating an inflammatory reaction is demonstrated by peroxidase double staining procedures. Inflammation in endomyocardial biopsies is diagnosed by the presence of ≥14 leukocytes/mm^2^.

Molecular analysis with DNA–RNA extraction and RT-PCR amplification of the viral genome should be mandatory [58]. In order to exclude systemic infection, peripheral blood should be investigated in parallel with endomyocardial biopsy [53, 58]; quantification of viral load and determination of viral replication may add diagnostic value [41, 53]. Primer pairs specific for coxsackievirus B, parvovirus B19 (PVB19), CMV, adenovirus type 2, influenza virus A, human herpesvirus 6 (HHV6), and EBV should be used to perform PCR and in the case of PVB19, quantitative real-time PCR.

As an innovative approach, next-generation sequencing (NGS) was recently evaluated for detecting potential pathogens of acute myocarditis from sera [59]. In this small investigation, virus-derived sequences were identified in seven of 17 cases, and the presence of viruses was confirmed by PCR or antigen testing in four patients.

## From infection to clinical scenarios

Infectious agents, including viruses such as entero-, cytomegalo-, and adenoviruses, bacteria such as *Borrelia burgdorferi* or *Chlamydia pneumonia*, protozoa, and even fungi can cause inflammatory heart disease leading to DCM. The most often identified infectious agents in DCM today are parvovirus B19, human herpes virus 6, and EBV. Many clinical faces of inflammatory heart disease coexist during different phases of the disease progression: Phase 1 is dominated by the viral infection itself, phase 2 by the onset of autoimmune reactions, and phase 3 by the progression to cardiac dilatation. In enteroviral myocarditis the infections phase was followed by an autoimmune phase, in which in humans both cellular- and antibody-mediated immune reactions played an important role. In this context we have previously also focused on the role of cytolytic antisarcolemmal and antimyolemma antibodies [60].

### Clinical scenarios

Several clinical scenarios can be appreciated from Table 1. The clinical phenotype should be assessed according to the patient’s symptoms (dyspnea, angina, arrhythmias, shock index). Standard laboratory parameters should also include markers of cardiac necrosis (creatinine kinase MB [CKMB], troponin I or T), of heart failure (N-terminal pro b‑type natriuretic peptide [NT-pro BNP] or BNP), of inflammation (leukocyte count and differential blood cell count, C‑reactive protein [CRP], or sedimentation rate). Standard 12-lead electrocardiography (ECG) and color flow echocardiography, the latter as a first-line imaging method, are obligatory. Cardiac magnetic resonance imaging (MRI) may be helpful to detect areas of inflammation or fibrosis by late gadolinium enhancement (LGE) and additional clues for inflammation when the Lake Louise Criteria are used. After exclusion of significant coronary artery disease (CAD) endomyocardial biopsy should performed. Its evaluation should include light microscopy with hematoxylin–eosin staining, PCR of microbial agents, particularly cardiotropic viruses, immunohistochemistry of the infiltrate and the leukocyte subpopulations, as well as immunofluorescent staining of cardiac structures in the biopsies and cardiac autoantibodies in the circulating blood. They are of possible diagnostic relevance if they fix complement and if they are found both in the blood and in the biopsy.

**Table 1:** From symptoms to a clinical syndrome to an etiological diagnosis in inflammatory dilated cardiomyopathy (DCMi)

Clinical phenotype	Grading of symptoms	Diagnostic features	Etiological diagnosis by EMB
Acute life-threatening heart failure, “fulminant myocarditis”, severe rhythm disturbance	Shock, NYHA III–IV, syncope	Elevated troponin I or T, and natriuretic peptides, abnormalities in ECG, echo, or MRI	Eosinophilic or toxic or giant cell myocarditis, Borreliosis, >50 infiltrating cells/mm^2^ by WHF, PCR on viral etiology in lymphocytic myocarditis variable
Acute heart failure	Dyspnea, edema, HFrEF. HFpEF, no CAD	Intermittent elevations of troponins and natriuretic peptides, abnormalities in ECG, echo, or MRI, anticardiac antibodies	Viral or autoreactive myocarditis or DCMi. PCR on viral etiology variable
Acute chest wall syndrome	Angina-like symptoms, but no CAD but possibly MINOCA	ÉCG with variable ST‑T alterations	Parvovirus B19 in EMB or other virus with or without pericarditis, inflammation optional
Chronic heart failure	Heart failure symptoms, HFrEF. HFpEF	Intermittent elevations of troponins and natriuretic peptides, abnormal ECG with LBBB, RBBB, AV-block, abnormal echo or MRI, anticardiac antibodies	Focal viral or nonviral (autoreactive)myocarditis with >14 cell/mm^2^
Chronic heart failure	Heart failure symptoms, HFrEF. HFpEF	Intermittent elevations of troponins and natriuretic peptides, abnormal ECG with LBBB, RBBB, AV-block, abnormal echo or MRI, anticardiac antibodies	No myocarditis (<14 cells/mm^2^), but persistence of parvovirus B19 genome

*AV* atrioventricular, *CAD* coronary artery disease, *DCMi* inflammatory dilated cardiomyopathy, *ECG* electrocardiogram, *EMB* endomyocardial biopsy, *HFpEF* heart failure with preserved ejection fraction, *HFrEF* heart failure with reduced ejection fraction, *LBBB* left bundle branch block, *RBBB* right bundle branch block, *MRI* magnetic resonance imaging, *echo* echocardiography, *NYHA* New York Heart Association, *PCR* polymerase chain reaction, *MINOCA* myocardial infarction with nonobstructive coronary arteries, *WHF* World Heart Federation criteria (Maisch et al. 1999 [61] and 2000 [62])

#### Fulminant myocarditis

This case came to our attention as expert witness in a civil court trial. A 25-year-old, previously healthy student of economics, who was formerly also an amateur rugby player, had moved from Hamburg to Berlin. His medical history was uneventful apart from minor attacks of asthma 5 years earlier and casual smoking. In early 2013 he suddenly complained about fatigue and shortness of breath during daily activities. His clinical odyssey started on February 18, when he consulted a physician for general medicine because of backpain and dyspnea, who transferred him to a specialist for orthopedics, who in turn did not find anything serious. But he did not pay attention to the symptom of dyspnea.

**Fig. 1:**
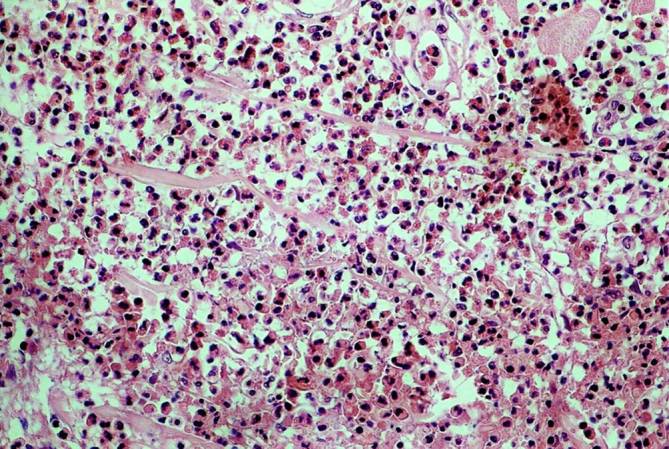
Postmortem analysis: H & E staining showing an abundance of eosinophils (×400)

The next day the patient complained about dysesthesia of both arms and shoulders and was transferred to the emergency department of a university hospital. There he was seen only by a neurologist, who neglected dyspnea and systemic manifestations and did not even examine the patient’s clinical status. He also omitted to draw blood or perform an ECG. At 17:45 h on the same day the patient collapsed at the threshold of his apartment. The emergency team attempted resuscitation but failed to do so. He was declared dead at 18:30 h. The parents requested a necropsy by forensic medicine, which made a diagnosis of single-organ vasculitis. We had to correct this diagnosis in court to fulminant eosinophilic pancarditis (Fig. 1).

The analysis for viral RNA and DNA by PCR was negative. Immunohistology showed some polyvalent immunoglobulin binding.

##### Lessons to be learned.

If a differential blood count with blood smear analysis along with an ECG and echocardiogram had been performed at the student’s first or second consultation, he would have been admitted immediately to an intensive care unit. Independently of a later endomyocardial biopsy, life-saving corticosteroid therapy could have been started on the basis of eosinophilia detected in the blood smear. Despite the impressive eosinophilic infiltrates at necropsy, the chance for survival would have been 70–80% at this stage and a most tragic outcome may have been avoided.

The diagnosis of fulminant myocarditis—to which belong eosinophilic heart disease, giant cell myocarditis sarcoidosis of the heart, and some very acute forms of lymphocytic myocarditis and cardiac inflammation by drugs such as the novel checkpoint inhibitors belong—is obviously a clinical syndrome, not an etiological diagnosis. It is a “diagnosis in search of its aetiology but with therapeutic options” [63]. A new American Heart Association statement in 2020 [64], recent European publications [2, 63, 65], and a Chinese expert consensus statement [66] underline the importance of symptomatic and supportive treatment including mechanical circulatory support (Impella microaxial pump and/or extracorporeal membrane oxygenation [ECMO]) as well as treatment of heart failure and cardiogenic shock but also of antiviral or intravenous immunoglobulin (IVIg) therapy whenever appropriate. Dose recommendations for IVIgG range between 20–40 g/day for 2 days and 10–20 g daily for 5–7 days [66], for IVIgM (Pentaglobin) 15–20 g on day 1 and day 3 [2, 67–69].

#### Acute parvovirus B19 myocarditis and vasculitis

**Fig. 2:**
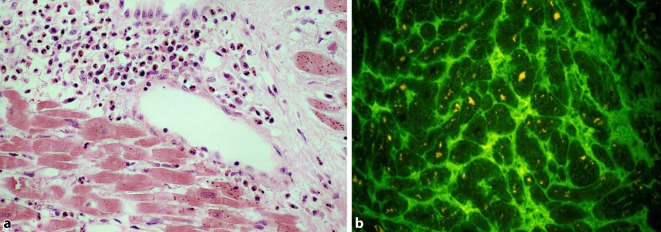
**a** Light microscopy demonstrates infiltrating lymphocytes, granulocytes, and some eosinophils around a small vessel. **b** Immunohistology: IgG binding to sarcolemma and vascular endothelium

**Fig. 3:**
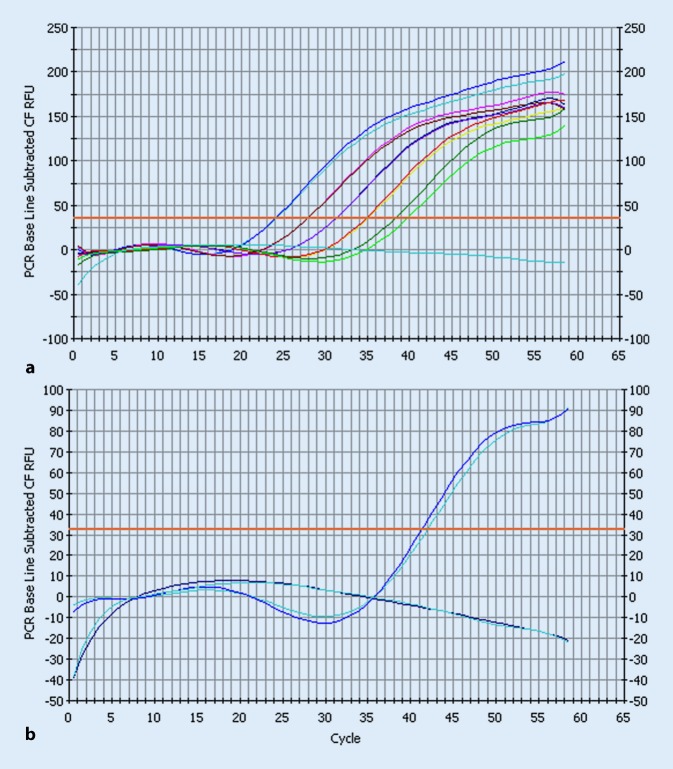
**a** Virus titration with Q‑PCR (10^8^–10^4^); **b** viral load: 7916 = 3.3 × 10^4^ copies/µg DNA

A 47-year-old male teacher was admitted to our hospital for precordial discomfort and dyspnea independent of physical exercise. His ECG showed a horizontal ST-segment depression of 1 mm from leads V_2_ to V_6_ at rest and a further depression of 2 mm in the anterolateral leads V_4_ to V_6_ at maximum exercise. In his echocardiogram a left ventricular enlargement with an end-diastolic diameter of 59 mm and a shortening fraction of 21% were noted. High-sensitivity troponin T was elevated at 0.051 ng/ml, CKMB was still normal. The level of CRP was increased (6.8 mg/dl), and the leukocyte count was 10,500/dl. We suspected unstable angina and anterior wall ischemia. However, coronary angiography did not show any coronary obstruction. Instead, left ventricular endomyocardial biopsy demonstrated a focal, mixed infiltrate around a small vessel with 21 cells/mm^2^ (Fig. 2a) and IgG- and IgM-binding to the vascular endothelium (Fig. 2b). A PCR of cardiotropic viruses was positive for parvovirus B19 virus with a high viral load of 3.3 × 10^4^ copies/µg DNA (Fig. 3). His diagnosis was parvovirus B19-positive inflammatory cardiomyopathy.

Heart failure treatment was initiated immediately and specific therapy with 15 g Pentaglobin on day 1 and 3 was administered after the biopsy results were available. The patient improved clinically during the following 4 weeks. His follow-up biopsy after 6 months demonstrated the eradication of inflammation, and a significant reduction of the viral load to only 19 copies/µg DNA. During follow-up, left ventricular function and diameters normalized.

##### Lesson learned.

**Fig. 4:**
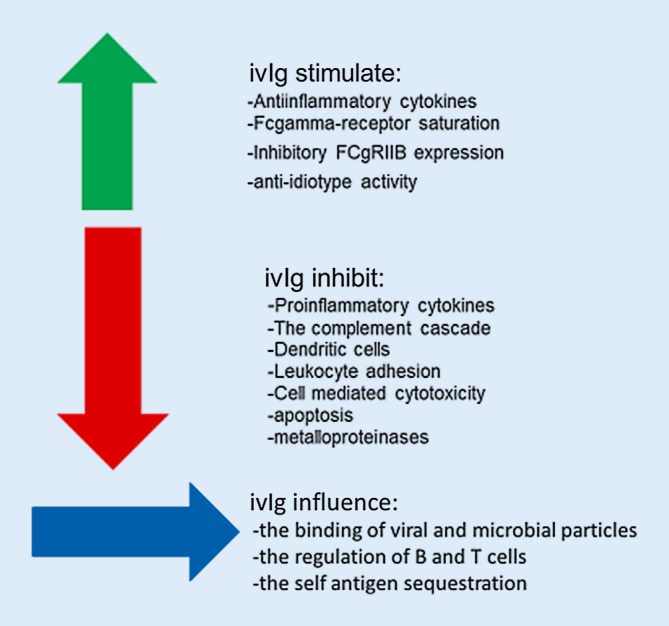
Effects of intravenous immunoglobulin (*ivIg*; IgGMA) treatment

Symptoms of parvovirus B19-positive myocarditis and vasculitis can mimic coronary artery disease. But the primary cause of angina is small vessel disease. The VP1u receptor of parvovirus B19 is located on erythroid progenitor cells such as permissive endothelial cells [70]. Anthony et al. [71] have shown that the anti-inflammatory activity of monomeric IgG is dependent on the sialylation of the *N*-linked glycan of the IgG Fc fragment. This explains a part of the anti-inflammatory action of IVIg. The IgM fraction of intravenous immunoglobulins with IgG, IgA, and IgM fractions (IVIgGAM) can play an additional role in controlling inflammatory and also autoimmune disease [2, 68]. Intravenous immunoglobulins interact widely with the host immune system. The wide spectrum of activities unfolded by IVIg can be appreciated in Fig. 4.

#### DCM with or without parvovirus B19 persistence: how to treat?

The third scenario reflects the overview of PB19 viral persistence without inflammation and compares it with virus-negative patients, who were biopsied for suspected myocarditis. These subgroups of patients were already described in 2003 [32] in a smaller study of 110 patients. The series has been expanded since then over the following years to a registry of 1098 DCM patients with inflammation and 2247 patients without inflammation (Table 2).

**Table 2:** Association of inflammation, ejection fraction (EF), and etiology (modified from [2])

	Inflammation EF >45%	Inflammation EF <45%	No inflammation EF >45%	No inflammation EF <45%
Number of patients	816	282	1663	584
Virus negative (%)	72.2	57.9	71.5	79.8
Parvovirus B19 positive (%)	20.4	33.3	23.9	17.6
Other viruses (%)	7.4	8.8	4.6	2.6

The following considerations can be derived from these registry data:

- In a large multicenter registry of biopsied patients with DCM, the proportion with myocardial inflammation was only one third.
- The association with parvovirus B19 was greatest in inflammatory cardiomyopathy with the greatest hemodynamic compromise (EF <45%).
- In DCM patients without inflammation, the parvovirus B19 genome was also detected in 17.6–23.9% of cases. It remains to be clarified whether this represents silent infection without inflammatory activity.

The therapeutic recommendation in the virus-positive patients with inflammation was IVIg with IgG, IgA, and IgM fractions (Pentaglobin). The weight-dependent dosages were 10–15 g on days 1 and 3. In the virus-negative subgroup with inflammation it was a combination of prednisolone (Decortin H) and azathioprine (Imurek) in a predefined dose for 6 months in the double-blind randomized ESETCID trial [72] or open-label treatment with the regimen published in the TIMIC study [73].

##### Lesson to be learned.

The major microbial agent in inflammatory cardiomyopathy is parvovirus B19 after an epidemiologic shift in the years 1990 to 1994 [2]. Intravenous Ig can eliminate infiltrates, as in case 2, also after infection with parvovirus B19. However, as the small studies and case analysis of our registry showed, it can eradicate entero- and adenovirus completely. In patients with a positive biopsy for parvovirus B19 intravenous treatment will only lead to a reduction in the percentage of patients with a significant load of parvovirus B19 but not eradicate the virus in all patients.

#### Immunosuppressive treatment in autoreactive myocarditis

The fourth scenario deals with DCM patients with an autoreactive form of myocardial inflammation. A substantial number of randomized and controlled or registry studies have examined the effect of immunosuppression in patients with DCM or suspected myocarditis [73–78]. In the randomized Myocarditis Trial of 1995 by Mason et al. [74], the overall result was “no benefit, no harm.” But in this trial the biopsies were not examined to exclude viral infection of the myocardium [75]. To date, immunosuppression is contraindicated in viral myocarditis according to current guidelines. In the TIMIC study, virus was excluded by PCR. Only severely compromised myocarditis patients without demonstrable viral persistence in the biopsy could be included in this double-blind randomized trial [73]. The ejection fraction in the treatment arm comprising 43 patients increased from 26.5% at baseline to 45.6% after 6 months (*p* < 0.001). Accordingly, left ventricular end-diastolic volume, left ventricular end-diastolic diameter, and New York Heart Association class decreased significantly [73].

The ESETCID (*E*uropean *S*tudy on the *E*pidemiology and *T*reatment of *C*ardiac *I*nflammatory *D*isease) is a double-blind, randomized, placebo-controlled three-armed trial with prednisolone and azathioprine for autoreactive (virus-negative) DCMi in patients with an ejection fraction <45% at baseline. Its intermediate results from the immunosuppressive treatment arm demonstrated a positive trend in ejection fraction and major adverse cardiac events after 6 months of treatment and a significant benefit after 1 year of follow-up [72]. Remarkably, the control group without immunosuppressive treatment also showed some spontaneous resolution of the infiltrate.

## Conclusion

In suspected inflammatory dilated cardiomyopathy or myocarditis, to date, only endomyocardial biopsy allows for the differentiation of virus-positive or virus-negative patients. This differentiation offers causative treatment options beyond heart failure and antiarrhythmic or device therapy.
